# Detection of extra pulses in synthesized glottal area waveforms of dysphonic voices

**DOI:** 10.1016/j.bspc.2019.01.007

**Published:** 2019-04

**Authors:** P. Aichinger, F. Pernkopf, J. Schoentgen

**Affiliations:** a Division of Phoniatrics-Logopedics, Department of Otorhinolaryngology, Medical University of Vienna, Waehringer Guertel 18-20, 1090, Vienna, Austria; b Signal Processing and Speech Communication Laboratory, Graz University of Technology, Inffeldgasse 16c/EG, 8010, Graz, Austria; c BEAMS (Bio-, Electro- And Mechanical Systems), Faculty of Applied Sciences, Université Libre de Bruxelles, 50, Av. F. D. Roosevelt, B-1050, Brussels, Belgium

**Keywords:** High-speed videolaryngoscopy, Glottal area waveforms, Extra pulses, Dysphonia, Modulation noise, Detection

## Abstract

**Background and objectives:**

The description of production kinematics of dysphonic voices plays an important role in the clinical care of voice disorders. However, high-speed videolaryngoscopy is not routinely used in clinical practice, partly because there is a lack of diagnostic markers that may be obtained from high-speed videos automatically. Aim of the study is to propose and test a procedure that automatically detects extra pulses, which may occur in voiced source signals of pathological voices in addition to cyclic pulses.

**Material and methods:**

Glottal area waveforms (GAW) are synthesized and used to test a detector for extra pulses. Regarding synthesis, for each GAW a cyclic pulse train is mixed with an extra pulse train, and additive noise. The cyclic pulse trains are varied across GAWs in terms of fundamental frequency, pulse shape, and modulation noise, i.e., jitter and shimmer. The extra pulse trains are varied across GAWs in terms of the height of the extra pulses, and their rates of occurrence. The energy level of the additive noise is also varied. Regarding detection, first, the fundamental frequency is estimated jointly with the cyclic pulse train waveform, second, the modulation noise is estimated, and finally the extra pulse train waveform is estimated. Two versions of the detector are compared, i.e., one that parameterizes the shapes of the cyclic pulses, and one that uses unparameterized pulse shape estimates. Two corpora are used for testing, i.e., one with 100 GAWs containing random extra pulses, and one with 25 GAWs containing extra pulses in the closed phases of each glottal phase representing subharmonic voices.

**Results and discussion:**

With pulse shape parameterization (PSP) a maximum mean accuracy of 88.3% is achieved when detecting random extra pulses. Without PSP, the maximum mean accuracy reduces to 82.9%. Detection performance decreases if the energy level of additive noise is higher than −25 dB with respect to the energy of the cyclic pulse train, and if the irregularity strength exceeds 0.1. For bicyclic, i.e., subharmonic voices, the approach fails without PSP, whereas with PSP, a mean sensitivity of 87.4% is achieved for subharmonic voices.

**Conclusion:**

A synthesizer for GAWs containing extra pulses, and a detector for extra pulses are proposed. With PSP, favorable detector performance is observed for not too high levels of additive noise and irregularity strengths. In signals with high noise levels, the detector without PSP outperforms the other one. Detection of extra pulses fails if irregularity strength is large. For subharmonic voices PSP must be used.

## 1 Introduction

The description of voice production kinematics plays an important role in the clinical care of dysphonic voices, because it aids the indication, selection, evaluation, and optimization of clinical treatment techniques. In clinical routine, voice production kinematics are primarily assessed by means of stroboscopic imaging of the vocal fold vibration [1,2]. However, due to the limitation of the stroboscopic method, many abnormal phenomena in vocal fold vibration may be disguised. For example, one needs to assume in stroboscopy that inter-cyclic variation of phonation pulses is small, because the behaviour of stroboscopy with large inter-cyclic variation depends on many unexplored factors and is thus hardly predictable. In other words, a sequence of phonation pulses with similar shapes is required to produce a smooth stroboscopic video. This limitation relates to the well-established Nyquist-Shannon sampling theorem that requires a sampling frequency higher than twice the highest frequency of the signal [3]. Currently, stroboscopy is often used beyond this limitation, although high-speed videolaryngoscopy and kymographic imaging are capable of imaging subsequent pulses with different shapes.

The pathophysiological process of extra pulsing is explained as follows. Extra pulsing may be caused by (slight) desynchronization of the anterior and posterior part of the vocal folds. This is a vibration mode that can be understood as an intermediate stage between modal phonation and biphonation / diplophonia. In extrapulsing, one cyclic oscillator is dominant in terms of amplitude, while the other one is kind of “shooting in between” pulses, without being “strong enough” (yet) to generate a distinct second vibration frequency. In the extreme case of extrapulsing that is known as double pulsing / alternating pulses, an extra pulse occurs in each and every quasi-closed phase of the cyclic pulses.

The occurrence of extra pulses in dysphonic voices is interesting from several viewpoints. First, the prevalence of such extra pulses in dysphonic voices is unknown, most likely because (1) stroboscopic imaging does not suffice to find extra pulses, and (2) it is labour intensive to manually search out extra pulses in high-speed videos or kymograms if lots of data needs to be analysed. Thus, extra pulses may often be overlooked in clinical practice.

With regard to the representation of extra pulses in kymographic imaging, it appears to be necessary to distinguish between videokymography (VKG), and digital kymography (DKG) [4]. In VKG, kymographic images are created in real time during endoscopic examination. Kymographic images of a chosen length are shown and updated with a rate reciprocal to its length. Usually, a length of 40 ms is chosen which results in an update rate of 25 Hz. If random extra pulses occur, these are visible for 40 ms only, and are thus hardly detectable visually. If extra pulses occur in a structured way, e.g., as approximately equally shaped extra pulses in each and every cycle, they can be seen easily. In DKG, kymographic images are created after recording. Given a vocal frequency of, e.g., 100 or 200 Hz, a 2 s phonatory segment includes 200 or 400 cycles. One needs to visually search out for extra pulses that may occur randomly in each of these cycles. Such a search is a tedious endeavour.

Second, extra pulses disturb substantially the harmonic spectrum of the voice sound, thus a significant auditory impact is expected from adding extra pulses to the cyclic pulse train of normal phonation. However, not much is known regarding the auditory attributes that a listener may assign to a voice sample containing extra pulses. Thus, extra pulses may often be overheard in clinical practice. In a past case study, the concept of “tonal raspiness” was proposed, which accounts for the pitch / tonality that is provoked by the cyclic pulse train, and the raspy component that is provoked by the extra pulse train [5]. This perspective agrees with Bregman’s well-established theory of auditory stream segregation [6]. We hypothesize that auditory raspiness is provoked by frequently occurring extra pulses, while unfrequently occurring extra pulses provoke auditory crackling [7]. Once a synthesizer for voices with extra pulses is available, the auditory impact of extra pulses on the voice sound can be investigated.

Third, the occurrence of extra pulses is likely to be triggered by mechanic and aerodynamic properties of the vocal folds and the phonatory process. These properties may be subject to clinical treatment (logopedic or surgical), thus it appears to be plausible that treatment may be more target oriented in cases for which extra pulses were identified. Finally, from a signal processing perspective, the proposals that we make may also be applicable in the future to other types of signals in which a cyclic pulse train is mixed with a random extra pulse train.

To the best of our knowledge, we present the first attempt towards automatic detection of random extra glottal pulses that may occur during quasi-closed phases of the normally occurring cyclic pulse train. A limitation of laryngeal high-speed videoendoscopy and kymography is that a lot of manual post-processing is required before a diagnostic marker can be displayed to a medical doctor, which impedes clinical acceptability of the approach. Thus, we explore here an approach towards the automatic appraisal of glottal area waveforms (GAW), which is intended to decrease the amount of manual labour required to obtain an underexplored diagnostic marker, i.e., a marker indicating the presence of extra pulses. We propose and test a method for the detection of extra pulses that occur during quasi-closed phases of random glottal cycles. The aim of this study is to further test and improve the detector that was proposed in the past [5]. The remainder of this article is structured as followed. In Section 2 we present related work. In Sections 3.1 and 3.2, the synthesis of the GAWs is explained. In Section 3.3, the detector architecture is explained. A simple version of the detector is compared to an advanced version that uses PSP for the estimation of the cyclic pulse train component. In Section 4, results regarding the detector performance are presented for different levels of additive noise and irregularity strengths, i.e., the detector is tested for robustness. Favorable performance is observed with PSP for low levels of additive noise and small irregularity. In signals with high noise levels, the detector without PSP outperforms the other one. For bicyclic signals / bigeminism / subharmonics PSP must be used. Detection of extra pulses fails for strongly irregular signals. In Section 5, conclusions are drawn and advices for the practical use of the detector are given.

## 2 Related work

In [8] several criteria to visually judge kymographic images of vocal fold vibration are presented. Examples for “cycle aberrations” are depicted in Fig. 7 of [8]. So-called “ripples” and “doubled medial peaks” are depicted in kymograms B and D. These descriptive attributes correspond to extra pulses that occur during the open phase of the phonatory cycle. In the depicted examples, ripples and double medial peaks occur regularly in each phonatory cycle. Also, a concept “large cycle-to-cycle variability” was used. Both “cycle aberrations” and “large cycle-to-cycle variability” are superordinate concepts to the extra pulses that we are investigating.

Fraj et al. [9] developed a synthesizer for pathological voices that uses a nonlinear wave-shaping model of the glottal area. The Klatt concatenated-curve model is used as a glottal area template [10], and modulation noise is simulated via polynomial distortion. The instantaneous frequency and a harmonic driving function are control parameters of the synthesizer. These parameters enable control of the pitch, amplitude, harmonic richness, open quotient, and irregularity by means of modulation noise. Regarding cycle length modulation noise, jitter and tremor are distinguished. Jitter is simulated as a two-point stochastic process added to the instantaneous phase on a sample-by-sample basis. Tremor is simulated as a band-pass filtered white Gaussian noise further added to the instantaneous phase. Amplitude modulation noise, i.e., shimmer, is only contained in the speech signal and not in the GAW. It results from vocal tract filtering of the source signal that contains jitter and tremor. It was shown that this synthesizer is capable of producing naturally sounding samples of dysphonic voices. As a complement to the work by Fraj et al., we propose to control and estimate cycle length and amplitude modulation noise via the modulation of individual pulses’ timings and heights at cycle-synchronous supporting points. This enables control and estimation of the modulation noise on a cycle-by-cycle basis instead on a sample-by-sample basis. The advantages of our approach compared to Fraj et al. are the following. First, our jitter is not a two-point process. Instead, the pulses of the cyclic pulse train may be anticipated or delayed with our approach by an arbitrary amount, and pulse shapes are time-warped accordingly to retain a smooth instantaneous phase. Second, our approach enables the estimation of modulation noise time series from observed signals. Finally, the bandwidth of our jitter does not depend on the sampling frequency.

Chen et al. [11] proposed a voice source model that models pulses of GAWs observed in three male and three female healthy subjects with high-speed videolaryngoscopy. We use this pulse shape model in our work for the synthesis of GAWs, and also for PSP in the estimation of the cyclic pulse train. The model has five parameters, i.e., the cycle length, the open quotient, the asymmetry coefficient, accounting for differences of the opening and closing phases’ durations, and two additional shape parameters for the opening and closing phases, i.e., one steepness parameter for each of the phases. The steepness parameters can be understood as the speed of the opening and closing phases.

Ikuma et al. [12,13], proposed a model for GAWs of pathological vocal fold vibration which is similar to ours. They model GAWs as a sum of a harmonic signal, a deterministic nonharmonic signal, and a random nonharmonic signal. Their harmonic signal is from a Fourier synthesizer, their deterministic nonharmonic signal is a sum of sinusoids the frequencies of which are not harmonically related, and their random nonharmonic signal is zero-mean white Gaussian noise. It would be inefficient to model extra pulses with Ikuma et al.’s model because the extra pulses are neither synthesizable with a reasonably small number of nonharmonic sinuses, nor are they zero-mean white Gaussian.

Randomly triggered extra pulses during quasi-closed phases of cyclic glottal pulses were observed in the past in a clinical case study of a dysphonic voice that sounded tonal and raspy [5]. A prototype for the detector was proposed, which identified correctly six observed extra pulses, and only one false alarm occurred. In this work, we further improve and test the detector that was proposed in the past.

## 3 Materials and methods

This section explains the synthesis of the GAWs, the detection of the extra pulses, as well as the performance measures and statistical analysis.

### 3.1 Synthesis of glottal area waveforms with random extra pulses

One-hundred GAWs are synthesized at a sampling frequency *f_s_* = 48 *kHz* with a length of 0.3 s. The synthesis of the GAWs involves the synthesis of the cyclic pulse train *d*_1_(*n*), and the synthesis of the extra pulse train *d*_2_(*n*), where *n* is the discrete time index. The synthesized GAW *d*′(*n*) = *d*_1_(*n*) + *d*_2_(*n*) + *η*(*n*), where *η*(*n*) is zero-mean white Gaussian noise. This signal model is adapted from [5]. In particular, control parameters are made explicit here.

**Fig. 1:**
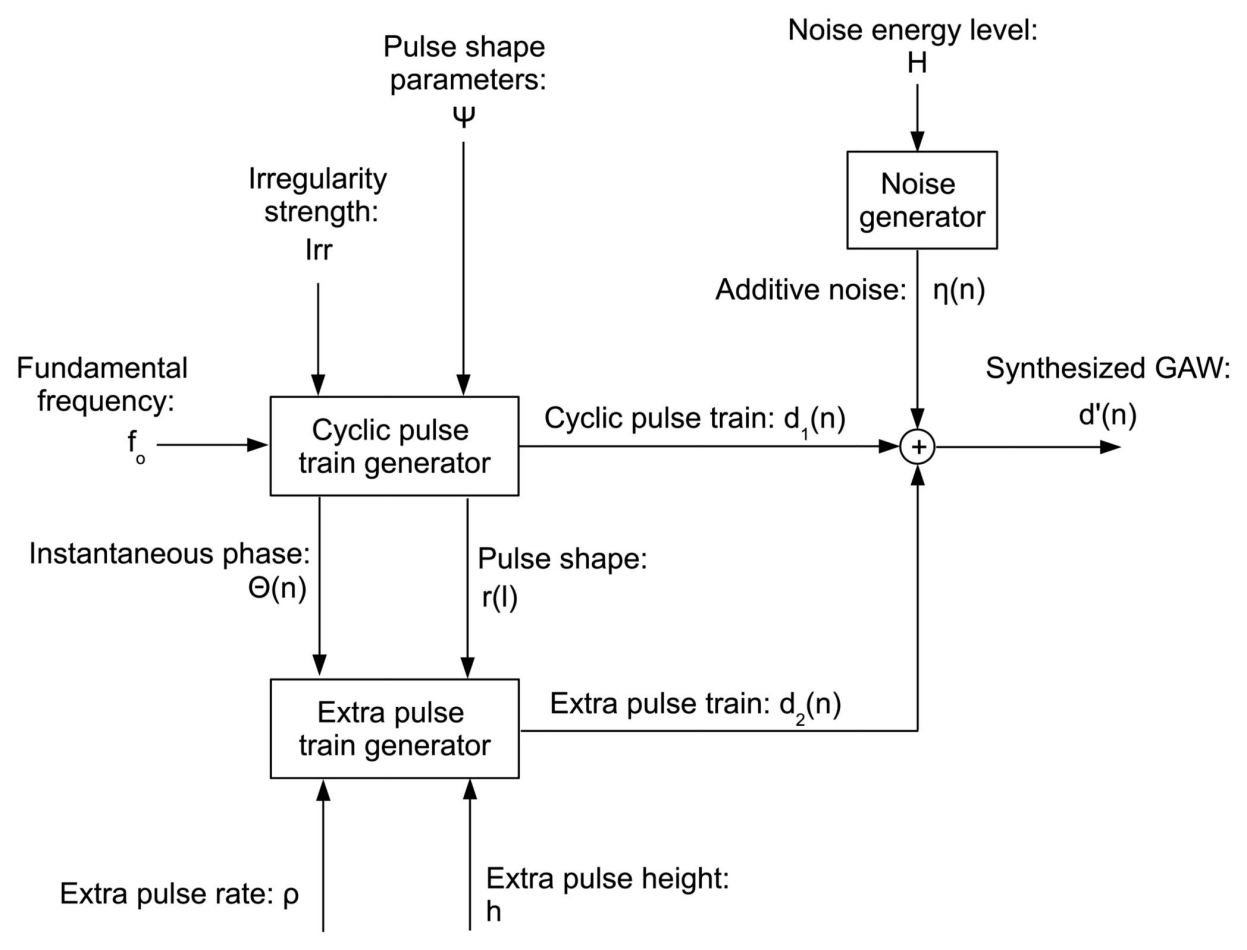
Overview block diagram of the GAW synthesizer. The GAW *d*’(*n*) is synthesized as a summation of a cyclic pulse train *d*_1_(*n*), an extra pulse train *d*_2_(*n*), and additive noise (*n*). The control parameters regarding the cyclic pulse train are the fundamental frequency *f_o_*, the irregularity strength *Irr*, and the pulse shape parameters Ψ. The control parameters regarding the extra pulse train are the extra pulse rate *ρ*, and the extra pulse height *h*. The control parameter regarding additive noise is the noise energy level *H*.

Fig. 1 shows the overview block diagram of the synthesizer. The fundamental frequency *f*_0_, the irregularity strength *Irr*, and the pulse shape parameters *Ψ* are input to the cyclic pulse train generator that puts out the cyclic pulse train *d*_1_(*n*), the instantaneous phase *Θ*(*n*), and the pulse shape *r*(*l*), where *l* is the cycle-relative discrete time index. The instantaneous phase *Θ*(*n*), the pulse shape *r*(*l*), the extra pulse rate *ρ*, and the extra pulse height *h* are input to the extra pulse train generator. The root mean square (RMS) energy level of the zero-mean white Gaussian noise *η*(*n*) is $H=20\cdot {\text{log}}_{10}\left(\sqrt{\bar{\eta {\left(n\right)}^{2}}}/\sqrt{\bar{d_{1}{\left(n\right)}^{2}}}\right).$ It is relative to the RMS energylevel of the cyclic pulse train *d*_1_(*n*), and given in dB.

**Fig. 2:**
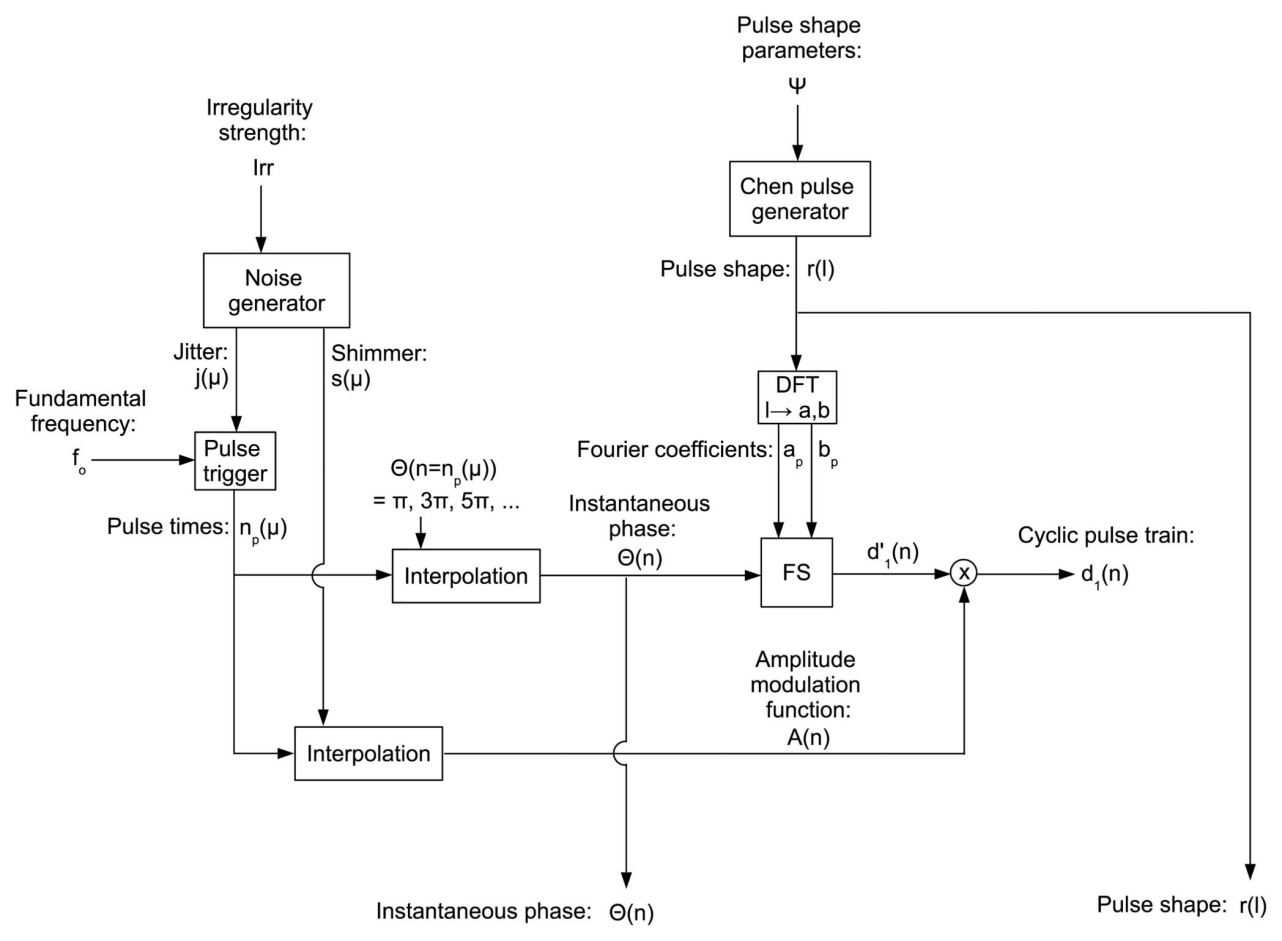
Block diagram of the cyclic pulse train generator. A pulse times vector *n_p_*(*μ*) is obtained owing to a fundamental frequency *f*_0_, and a cycle length modulation noise vector *j*(*μ*), controlled by irregularity strength *Irr*. An amplitude modulation noise vector *s*(*μ*) is also obtained. The instantaneous phase *Θ*(*n*) and the amplitude modulation function *A*(*n*) are obtained by interpolation. The pulse shape *r*(*l*) is a Chen pulse [11], controlled by parameters *Ψ*. The Fourier coefficients *a_p_* and *b_p_* of (*l*), and *Θ*(*n*) are input to a Fourier synthesizer (FS). Its output *d*′_1_ (*n*) is multiplied by *A*(*n*) to obtain the cyclic pulse train *d*_1_(*n*).

**Fig. 3:**
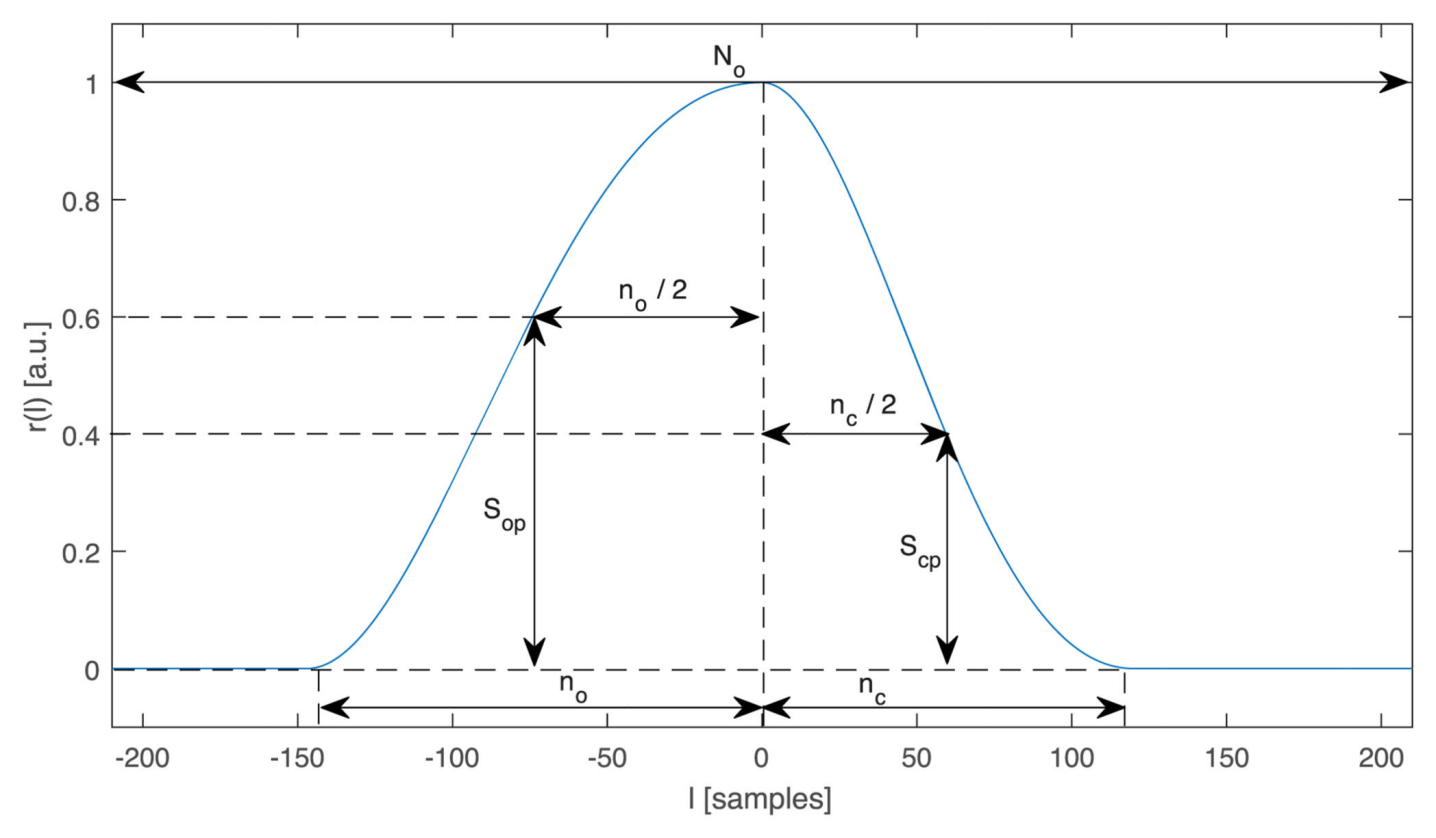
Example of a pulse shape, generate with the Chen model [11]. The parameters used in this example are the fundamental frequency *f_o_* = 115 *Hz*, the open quotient *OQ* = 0.64, the asymmetry parameter *α* = 0.55, the opening speed *S_op_* = 0.6, and the closing speed *S_cp_* = 0.4. The cycle length in samples is rounded to the nearest even integer, i.e., $N_{0}^{even}=\left\lfloor (f_{s}/f_{0}+1)/2\right\rfloor \cdot 2,$ the sampling frequency *f_s_* = 48 *kHz*, the length of the opening phase $n_{o}=\alpha \cdot OQ\cdot N_{0}^{even},$ and the length of the closing phase $n_{c}=OQ\cdot N_{0}^{even}-n_{o}.$ The crossings *r*(*l*) = *S_cp_* and *r*(*l*) = *S_op_* temporally halve the opening phase and the closing phase. *S_op_* and *S_cp_* are shape parameters of the opening and closing. The pulse is centred such that *argmax*(*r*(*l*)) = 0.

Fig. 2 shows the block diagram of the cyclic pulse train generator. The cyclic pulse train *d*_1_(*n*) is obtained as follows. First, the instantaneous phase *Θ*(n) is obtained. Therefore, the pulse times *n_p_*(*μ*) = *μ* · *N*_o_ + *j*(*μ*), where *μ* ∈ ℤ is the pulse index, the cycle length in samples *N*_o_ = *f_s_*/*f*_o_, and *j*(*μ*) is the time shift of the *μ^th^* pulse. The cycle length modulation noise, i.e., jitter, is drawn from a Gaussian distribution, i.e., $j(\mu )˜𝒩\;(0,Irr\cdot N_{0}),$ where *Irr* is the irregularity strength, and 𝒩(μ,σ) denotes a Gaussian distribution with mean *μ* and standard deviation *σ*. The instantaneous phase at pulse locations *Θ*(*n* = *n_p_*(*μ*)) = *π* · Σ_*μ* ∈ ℤ_[2 · *μ* + 1], and is obtained between pulse locations via spline interpolation. Second, the amplitude modulation function *A*(*n*) is obtained at pulse locations *A*(*n* = *n_p_*(*μ*)) = *s*(*μ*), where *s*(*μ*) is the amplitude modulation noise, i.e., shimmer, which is drawn from a Gaussian distribution s(μ)˜𝒩 (1,Irr). Between pulse locations, *A*(*n*) is obtained by shape preserving cubic interpolation. Third, a pulse shape *r*(*l*) is obtained with a Chen pulse generator [11]. Fig. 3 shows an example of a pulse shape. The real part and imaginary part Fourier coefficients *a_p_* and *b_p_* are obtained by discrete Fourier transformation (DFT) of the pulse shape (*l*), where *p* is the partial index. Fourth, the cyclic pulse train *d*_1_(*n*) is obtained via Fourier synthesis taking *a_p_*, *b_p_*, and *Θ*(*n*) as inputs, i.e., $d\prime_{1}(n)=a_{0}+\sum_{p=1}^{30}[a_{p}\cdot \text{cos}\left(p\cdot \Theta (n)\right)+b_{p}\cdot \text{sin}\left(p\cdot \Theta (n)\right)],$ and amplitude modulation, i.e., *d*_1_(*n*) = *A*(*n*) · *d*′_1_(*n*). The number of partials is 30.

The extra pulse train *d*_2_(*n*) is obtained as follows. The trigger *ξ*(*μ*) of the extra pulses is drawn from a Bernoulli distribution, i.e., *ξ*(*μ*) ∈ {0, 1}, with the extra pulse rate *ρ* = *p*(*ξ* = 1). The extra pulse train *d*_2_(*n*) = *h* · ∑_*μ*_ *ξ*(*μ*) · *r_d_*(*l_d_*), where *r_d_*(*l_d_*) is the delayed version of *r*(*l*), with *l_d_* = *l* − *n_p_*(*μ*) · *f_s_* − *N*_0_/2, and *h* is the extra pulse height. To enable delay times that are not necessarily integer multiples of the sampling interval 1/*f_s_*, fractional delays are made available via piecewise cubic interpolation of *r*(*l*).

**Fig. 4:**
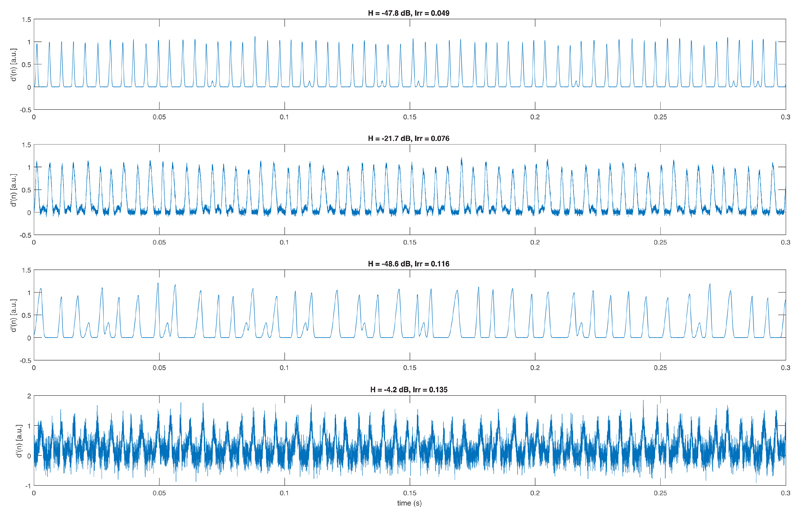
Examples of synthesized GAWs. The top subplot shows a GAW with a small level *H* of additive noise, and a small irregularity strength *Irr* (class I). Here, the extra pulses are clearly visible. The second subplot shows a GAW with an increased level *H* of additive noise, and a small irregularity strength *Irr* (class II). Here, extra pulses are less clearly visible. The third subplot shows a GAW with a small level *H* of additive noise, and a larger irregularity strength *Irr* (class III). The extra pulses are visible. The bottom subplot shows a GAW with a large level *H* of additive noise, and a large irregularity strength *Irr* (class IV). Extra pulses are not identifiable visually.

The time-invariant parameters *f_o_*, *Irr*, *H*, *ρ*, *h*, and *Ψ* = {*OQ*, *α*, *S_op_*, *S_cp_*} are random numbers drawn for each GAW from distributions defined in Table 1. Truncated normal distributions $𝒩(\mu ,\sigma^{2},x,y)$ and uniform distributions 𝒰(x,y) are used, where *μ* and *σ* are the means and standard deviations, and *x* and *y* are the lower and upper limits of the probability density functions (PDF). Further, the parameters *Irr* and *H* are balanced such that 25 GAWs are with parameters *H* ≤ −25 and *Irr* ≤ 0.1 (class I), 25 are with parameters *H* > −25 and *Irr* ≤ 0.1 (class II), 25 are with parameters *H* ≤ −25 and *Irr* > 0.1 (class III), and 25 are with parameters *H* > −25 and *Irr* > 0.1 (class IV). Fig. 4 shows example synthesized GAWs for each of the four classes.

### 3.2 Synthesis of bicyclic glottal area waveforms

In an additional experiment, twenty-five bicyclic GAWs are synthesized. The synthesizer described in the previous section is used with a fixed extra pulse rate *ρ* = 1. Setting the extra pulse rate to one results in the triggering of one extra pulse during the closed phase of each glottal cycle, and thus alternating patterns in the time domain (bigeminism). This signal type relates to a frequently occurring type of voice, i.e., subharmonic voice, which is characterized by alternating magnitudes of partials in the frequency domain. Only signals of class I are synthesized, i.e., the irregularity strength Irr=𝒰(0,0.1), and the energy level of the additive noise H=𝒰(−50,−25).

### 3.3 Detection of extra pulses

A detector for extra pulses is proposed in the following. It is based on parameter estimation and resynthesis of the GAWs under test. It is a composition of joint estimation of the fundamental frequency and the cyclic pulse train, estimation of the modulation noise, and modelling of the extra pulse train. Parts of the detector were proposed in the past [5]. The method is here improved by (1) the use of a parametric pulse shape model, i.e., the Chen pulse model [11], (2) the use of a new candidate selection procedure in the fundamental frequency extraction, and (3) a peak-picking free extra pulse train estimator. The method is described as follows.

#### 3.3.1 Joint estimation of the fundamental frequency and the cyclic pulse train waveform

**Fig. 5:**
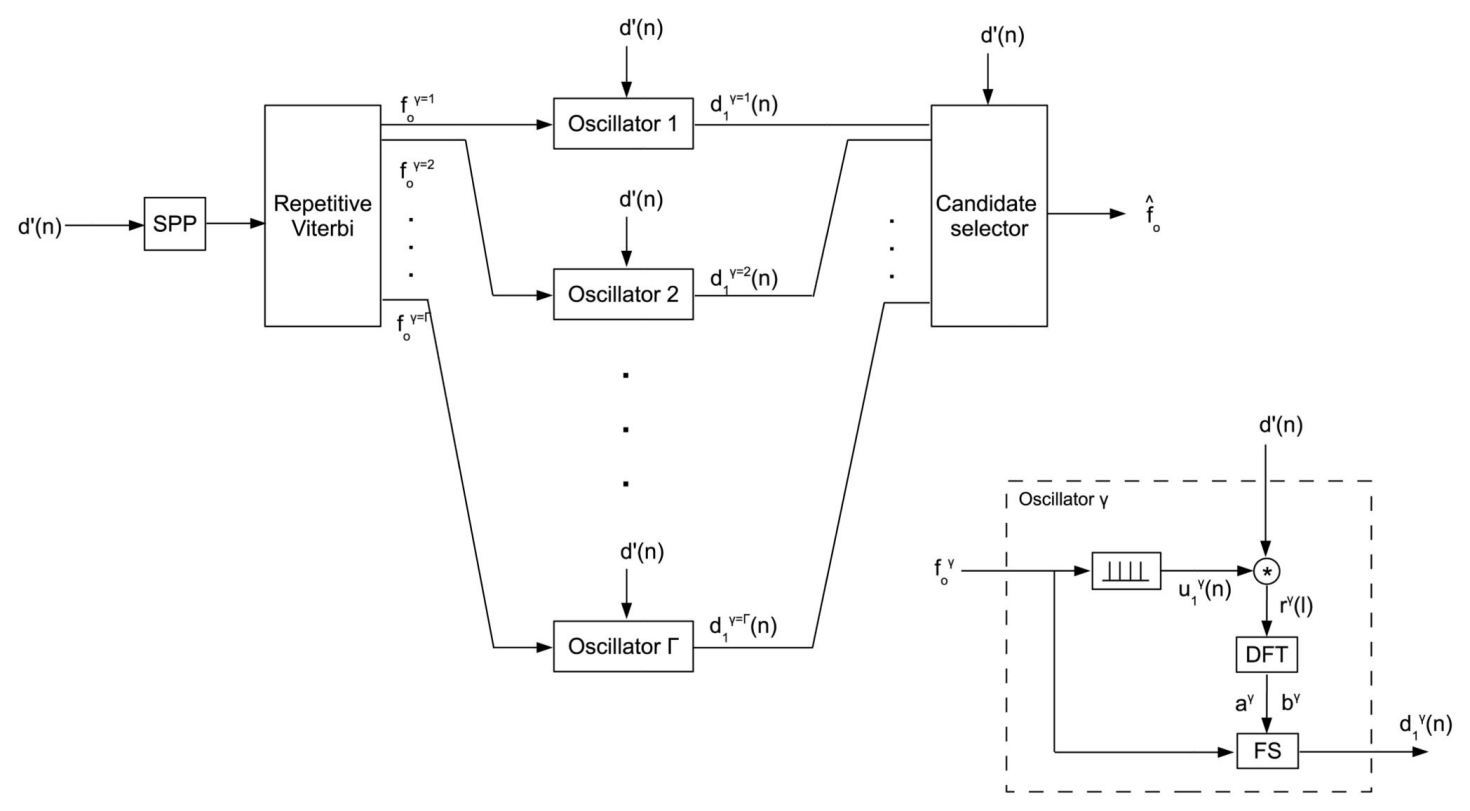
Block diagram of the fundamental frequency extractor. Fundamental frequency candidates $f_{0}^{\gamma }$ are obtained from the GAW *d*′(*n*) by spectral peak picking (SPP) and repetitive execution of the Viterbi algorithm (six times). For each fundamental frequency candidate $f_{0}^{\gamma }$ a cyclic pulse train candidate *d*_1_^*γ*^(*n*) is obtained by cross-correlating a unit-pulse train $u_{1}^{\gamma }(n)$ with the GAW ′(*n*), Fourier transformation of the cross-correlation vector, i.e., the pulse shape *r*^*γ*^(*l*), and Fourier synthesis (FS). Cyclic pulse train candidates *d*_1_^*γ*^(*n*) are added together owing to a candidate selection vector *S* = *s^*γ*^* ∈ {0, 1}. The cyclic pulse train estimate ${\hat{d}}_{1}(n)$ is subtracted from *d*′(*n*) to obtain *e*_1_(*n*), which is minimized with respect to *S*.

First, the fundamental frequency *f_o_* and the cyclic pulse train *d*_1_(*n*) are jointly estimated as shown in Fig. 5. The method is adapted from the one described in [14]. A 32 ms Hann window with a 16 ms overlap is used for blocking signals. Candidate *f_o_* -tracks $f_{o}^{\gamma }$ are obtained by picking peaks in the spectrum of the GAW *d*′(*n*), and applying the Viterbi algorithm six times, as in the “fast” setup described in [14]. The candidate index *γ* = 1, 2, …, *Γ*, and *Γ* is the number of candidates. No high-pass filtering is used, as was for the analysis of audio signals in [14]. Candidate cyclic unit pulse trains $u_{1}^{\gamma }(n)$ are created for each $f_{o}^{\gamma }.$ Candidate cyclic pulse shapes *r^γ^*(*l*) are obtained by cross-correlating candidate $u_{1}^{\gamma }(n)$ with the observed GAW *d*′(*n*). The candidate *f_o_* -tracks $f_{o}^{\gamma }$ and the pulse shapes’ discrete Fourier coefficients *a^γ^* and *b^γ^* are used in a Fourier synthesizer, which determines candidate cyclic pulse trains $d_{1}^{\gamma }(n).$ For further details the interested reader is referred to [14].

We propose “ultra fast” candidate selection that replaces the candidate selection approach described in [14]. The estimate of the cyclic pulse train *d*_1_(*n*) is given by ${\hat{d}}_{1}(n)=\sum_{\gamma =1}^{\Gamma }s^{\gamma }\cdot d_{1}^{\gamma }(n),$ where the binary candidate selection vector *S* = *s^γ^* ∈ {0, 1}, and *Γ* is the number of candidates. The optimal candidate selection vector *S_opt_* is chosen so as to minimize the RMS error $E_{1}=20\cdot {\text{log}}_{10}\left(\sqrt{\bar{e_{1}{\left(n\right)}^{2}}}/\sqrt{\bar{d\prime {\left(n\right)}^{2}}}\right)$ of the error waveform $e_{1}(n)=d\prime (n)-{\hat{d}}_{1}(n),$ i.e., *S_opt_* = *argmin*[*E*_1_(*S*)].

The candidates are sorted such that $d_{1}^{\gamma =1}(n)$ is the one with the largest signal energy and $d_{1}^{\gamma =\Gamma }(n)$ is the one with the smallest. The candidate selection vector *S* is initialized as a *Γ* -dimensional zero vector. For all candidate indices *γ* individually, the state of the *γ*^th^ element of *S* is switched. If candidates overlap temporally or if *E*_1_ does not decrease, the switch is reverted. The loop is repeated until convergence, i.e., until no improvement of *E*_1_ is observed for any switch of *s^γ^*. The fundamental frequency estimate ${\hat{f}}_{\text{o}}(t)=\left\{f_{\text{o}}^{\gamma }(t)\forall \gamma |s_{\text{o}pt}^{\gamma }=1\right\},$ where *t* is the block index, and $s_{opt}^{\gamma }$ are the elements of *S_opt_*.

#### 3.3.2 Modulation noise estimation

**Fig. 6:**
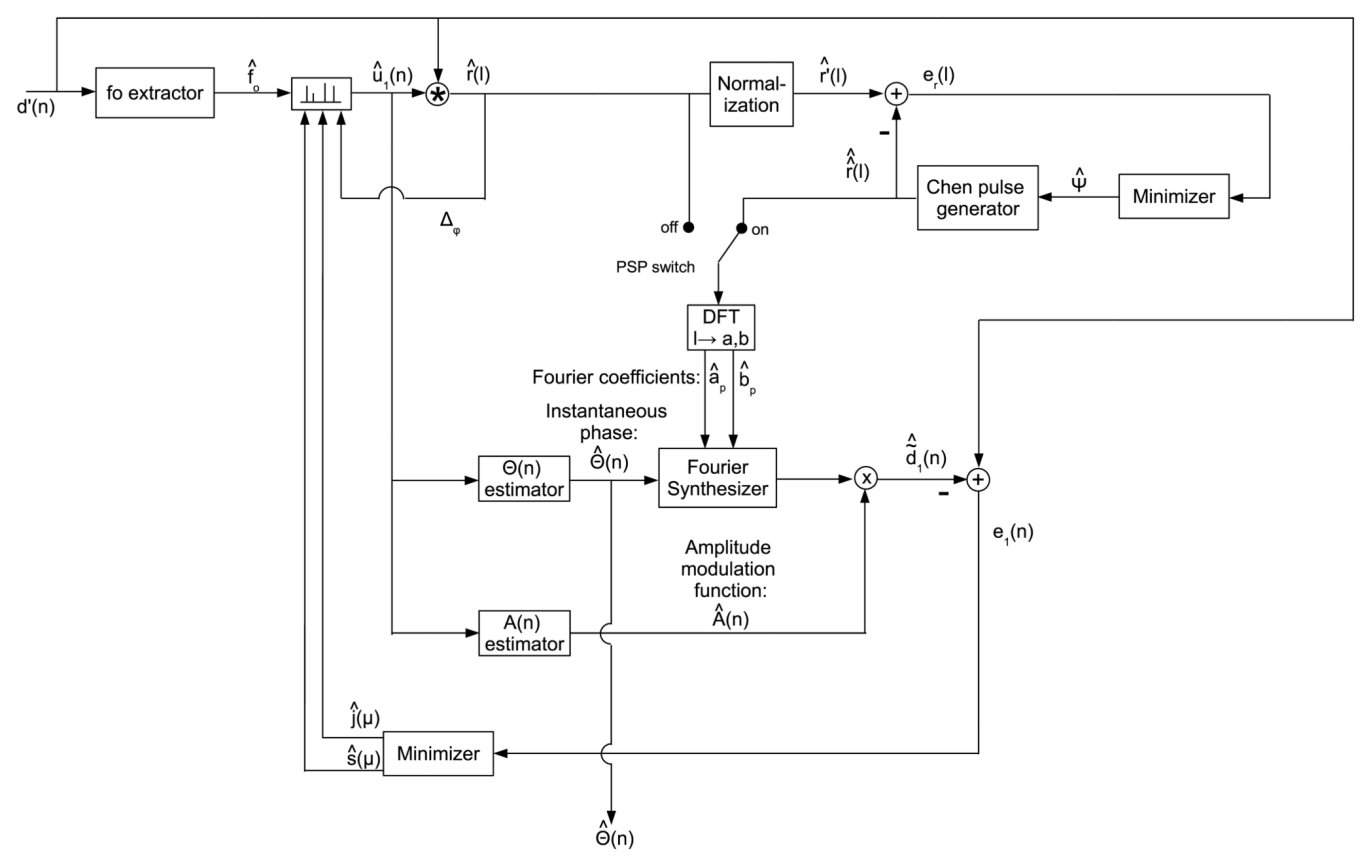
Block diagram regarding the estimation of the modulation noise. A quasi-unit pulse train ${\hat{u}}_{1}\left(n\right)$ is cross-correlated with GAW *d*′(*n*) to obtain the pulse shape estimate $\hat{r}\left(l\right).$ Via a pulse shape parameterization (PSP) switch, either $\hat{r}\left(l\right)$ or a parameterized version $\hat{\hat{r}}\left(l\right)$ is used. The parameterized pulse shape $\hat{\hat{r}}\left(l\right)$ is obtained from a Chen pulse generator, the control parameters $\hat{\Psi }$ of which are obtained via minimization of the parameterization error $e_{r}\left(l\right)=\hat{r}\prime \left(l\right)-\hat{\hat{r}}\left(l\right).$ The modulated cyclic pulse train ${\hat{\tilde{d}}}_{1}\left(n\right)$ is obtained with a Fourier synthesizer, taking the pulse shape’s Fourier coefficients ${\hat{a}}_{p}$ and ${\hat{b}}_{p},$ as well as the instantaneous phase estimate $\hat{\Theta }\left(n\right)$ as inputs. Its output is multiplied by the amplitude modulation function estimate $\hat{A}\left(n\right).$ The modulation noise vector estimates $\hat{j}\left(\mu \right)$ and $\hat{s}\left(\mu \right)$ perturb the quasi-unit pulse train ${\hat{u}}_{1}(n),$ and are obtained by minimizing the error ${\tilde{e}}_{1}\left(n\right)=d\prime \left(n\right)-{\hat{\tilde{d}}}_{1}\left(n\right).$

Second, the modulation noise is estimated as shown in Fig. 6. The method is adapted from [5]. In particular, we add here the option of PSP. A quasi-unit pulse train ${\hat{u}}_{1}(n)$ is cross-correlated with GAW *d*′(*n*) to obtain the pulse shape estimate $\hat{r}(l).$ Via a pulse shape parameterization (PSP) switch, either $\hat{r}(l)$ or a parameterized version $\hat{\hat{r}}(l)$ is used. The parameterized pulse shape $\hat{\hat{r}}(l)$ is obtained from a Chen pulse generator, the control parameters $\hat{\Psi }$ of which are obtained via minimization of the parameterization error $e_{r}(l)=\hat{r}\prime (l)-\hat{\hat{r}}(l),$ where $\hat{r}\prime (l)$ is a normalized version of $\hat{\hat{r}}(l).$ The modulated cyclic pulse train ${\hat{\tilde{d}}}_{1}(n)$ is obtained with a Fourier synthesizer, taking the pulse shape’s Fourier coefficients ${\hat{a}}_{p}$ and ${\hat{b}}_{p},$ as well as the instantaneous phase estimate $\hat{\Theta }(n)$ as inputs. Its output is multiplied by the amplitude modulation function estimate $\hat{A}(n).$ The modulation noise vector estimates $\hat{j}(\mu )$ and $\hat{s}(\mu )$ perturb the quasi-unit pulse train ${\hat{u}}_{1}(n),$ and are obtained by minimizing the error ${\tilde{e}}_{1}(n)=\hat{d}\prime (n)-{\hat{\tilde{d}}}_{1}(n).$

In more detail, the fundamental frequency estimate ${\hat{f}}_{o}$ drives a quasi-unit pulse oscillator providing ${\hat{u}}_{1}(n)=\sum_{\mu }\hat{s}(\mu )\cdot \delta \left[n-\mu \cdot {\hat{N}}_{0}-\hat{j}\left(\mu \right)-\Delta_{\phi }\right],$ where $\hat{s}\left(\mu \right)$ is the shimmer estimate, $\hat{j}\left(\mu \right)$ is the jitter estimate, ${\hat{N}}_{0}=\left\lfloor \left(f_{s}/{\hat{f}}_{0}+1\right)/2\right\rfloor \cdot 2$ is the cycle length estimate in samples rounded to the nearest even integer, and $\Delta_{\phi }=argmax\left(\hat{r}(l)\right)$ is a phase shift that aligns pulses of ${\hat{u}}_{1}\left(n\right)$ with the maxima of pulses of cyclic pulse train *d*_1_(*n*), and this centres $\hat{r}\left(l\right)$ such that *argmax* $\left(\hat{r}(l)\right)=0.\;{\hat{u}}_{1}(n)$ is cross-correlated with GAW *d*′(*n*) and normalized to obtain the pulse shape estimate $\hat{r}(l)=\frac{1}{\sum_{n}{\hat{u}}_{1}(n)}\cdot \sum_{n}{\hat{u}}_{1}(n)\cdot d\prime (n-l),$ where *l* goes from $-{\hat{N}}_{0}/2+1\;\text{to}\;{\hat{N}}_{0}/2-1.$ Thus, *û*_1_(*n*) is obtained recursively. The pulse shape estimate $\hat{r}(l)$ is parameterized with a Chen pulse model with parameters $\hat{\Psi }=\left\{\hat{OQ},\hat{\alpha },{\hat{S}}_{op},{\hat{S}}_{cp}\right\}$ as follows. The parameters are initialized as ${\hat{\Psi }}_{0}=\left\{0.5,0.5,0.5,0.5\right\}\cdot \;{\hat{r}}^{\prime }(l)$ is obtained by subtracting $\hat{\hat{r}}(l)$ from a normalized $\hat{r}\prime (l)$ Subequently, $\hat{r}\prime (l)$ is further normalized such that min $\left({\hat{r}}^{\prime }(l)\right)=0$ and max $\left({\hat{r}}^{\prime }(l)\right)=1.$ $\hat{\hat{r}}(l)$ is shifted in time such that its maximum coincides with the maximum of $\hat{r}\prime (l).$ The mean square model error is obtained as $E_{r}=\bar{e_{r}^{2}}(l).$ The parameters $\hat{OQ},\hat{\alpha },{\hat{S}}_{op},\;\text{and}\;{\hat{S}}_{cp}$ are iteratively optimized one by one by golden section search and parabolic interpolation to minimize *E_r_* [15,16]. Each parameter is constraint to the interval [0.1, 0.9]. Each step of iteration includes optimization of each parameter in the order $\hat{OQ},\hat{\alpha },{\hat{S}}_{op},\;\text{and}\;{\hat{S}}_{cp}.$ Estimation is stopped as soon as the improvement of *E_r_* decreases in the last iteration step below 10^−5^. Optionally, PSP is switched on and off. Accordingly, either the cross-correlation vector $\hat{r}(l)$ or its parameterized version $\hat{\hat{r}}(l)$ is used.

The instantaneous phase estimate $\hat{\Theta }(n)$ and the amplitude modulation function estimate $\hat{A}(n)$ are obtained from the pulse train estimate ${\hat{u}}_{1}(n).$ In particular, $\hat{\Theta }(n)=\pi \cdot \sum_{\mu \in \mathbb{Z}}\left[2\cdot \mu +1\right]$ at pulse locations of ${\hat{u}}_{1}(n),$ i.e., at $n=\mu \cdot {\hat{N}}_{0}+\hat{j}(\mu )+\Delta_{\phi },$ and spline interpolated in between, and $\hat{A}(n)=\hat{s}(\mu )$ at pulse locations of ${\hat{u}}_{1}(n),$ and obtained by shape preserving cubic interpolation in between.

The cyclic pulse train estimate ${\hat{d}}_{1}(n)$ is obtained via Fourier synthesis taking the pulse shape’s Fourier coefficients ${\hat{a}}_{p},{\hat{b}}_{p},\;\text{and}\;\hat{\Theta }\left(n\right)$ as inputs, and subsequent amplitude modulation, i.e., ${\hat{d}}_{1}^{\prime }\left(n\right)={\hat{a}}_{0}+\sum_{p=1}^{10}\left[{\hat{a}}_{p}\cdot \text{cos}\left(p\cdot \hat{\Theta }\left(n\right)\right)+{\hat{b}}_{p}\cdot \text{sin}\left(p\cdot \hat{\Theta }\left(n\right)\right)\right],\;\text{and}\;{\hat{d}}_{1}\left(n\right)=\hat{A}\left(n\right)\cdot {\hat{d}}_{1}^{\prime }\left(n\right).$ The number of partials is 10.

The jitter and shimmer vector estimates $\hat{j}\left(\mu \right)$ and $\hat{s}\left(\mu \right)$ are obtained via minimizing the RMS error $E_{1}=20\cdot {\text{log}}_{10}\left\{\sqrt{\bar{e_{1}^{2}(n)}}/\sqrt{\bar{{\hat{d}}_{1}^{2}(n)}}\right\},$ i.e., $\left[\hat{j}\left(\mu \right),\hat{s}\left(\mu \right)\right]={\text{argmin}}_{j\left(\mu \right),s\left(\mu \right)}\left\{E_{1}\left(j\left(\mu \right),s\left(\mu \right)\right)\right\}.$ The interior-point algorithm is used for each pulse individually [17,18]. After the last pulse, the procedure iteratively refines the estimate until convergence, i.e., until the model error improvement cumulated from the first to the last pulse decreases below 0.01 dB.

#### 3.3.3 Extra pulse train waveform estimation

**Fig. 7:**
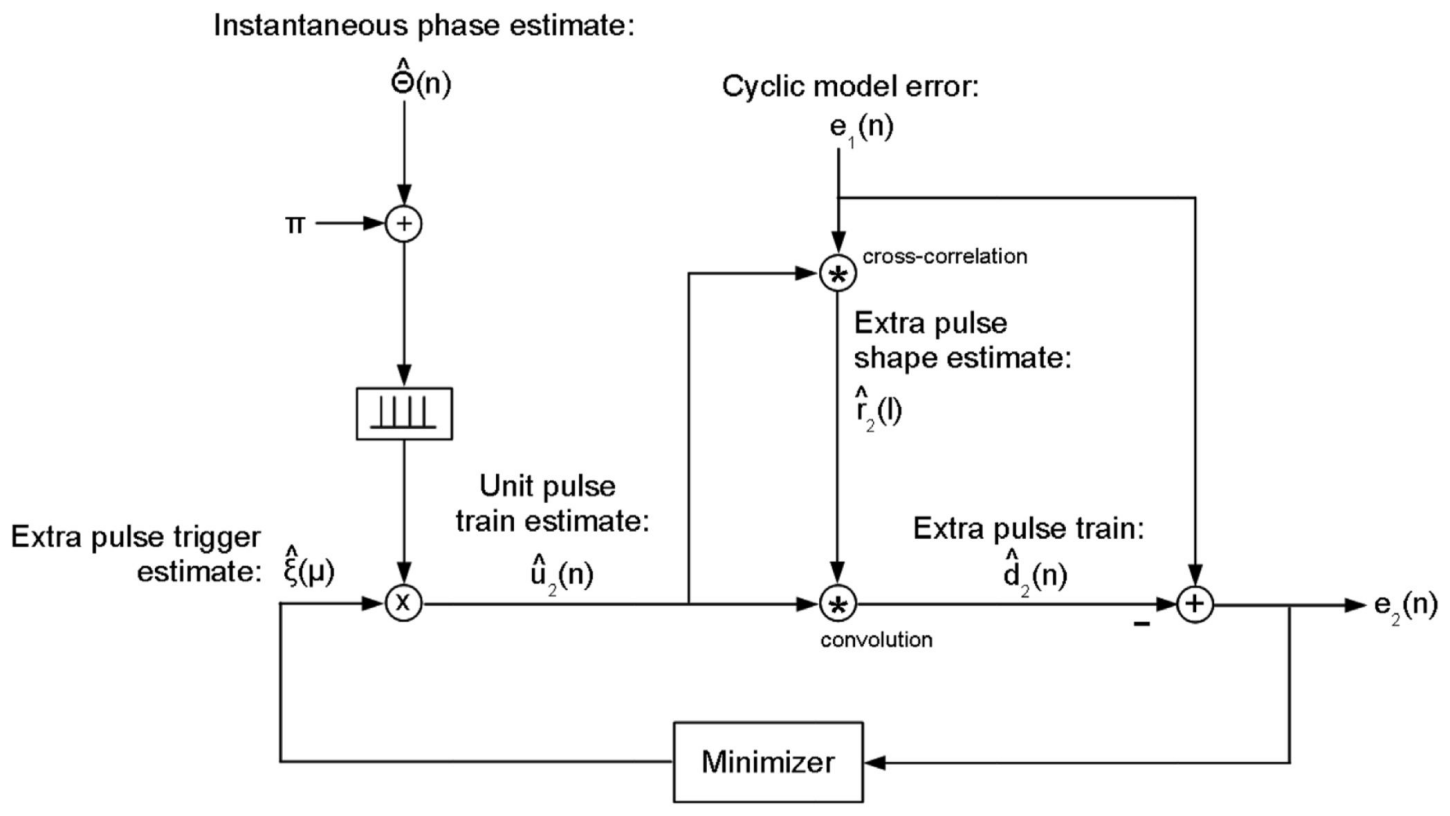
Block diagram regarding the estimation of the extra pulse train. The constant phase shift *π* is added to the instantaneous phase estimate $\hat{\Theta }(n),$ to obtain an extra unit pulse train estimate ${\hat{u}}_{2}(n).$ ${\hat{u}}_{2}(n)$ is cross-correlated with the error *e*_1_(*n*) of the cyclic model to obtain the extra pulse shape ${\hat{r}}_{2}(l).$ The extra pulse train estimate ${\hat{d}}_{2}(n)$ is obtained by convolving ${\hat{u}}_{2}(n)$ with ${\hat{r}}_{2}(l).$ The extra pulse trigger estimate $\hat{\xi }(\mu )\in \left\{0,1\right\}$ is obtained via minimizing the model error $e_{2}(n)=e_{1}(n)-{\hat{d}}_{2}(n).$

Finally, the extra pulse train estimate ${\hat{d}}_{2}(n)$ is obtained as shown in Fig. 7. An *M* -dimensional binary candidate selection vector $\hat{\Xi }=\hat{\xi }(\mu )\in \left\{0,1\right\},$ where *M* is the number of pulses in the cyclic pulse train estimate ${\hat{d}}_{1}(n).$ The optimal candidate selection vector ${\hat{\Xi }}_{opt}={\hat{\xi }}_{opt}(\mu )$ is chosen by minimizing the RMS error $E_{2}=20\cdot {\text{log}}_{10}\left(\sqrt{\bar{e_{2}{\left(n\right)}^{2}}}/\sqrt{\bar{d^{\prime }{\left(n\right)}^{2}}}\right)$ of the error waveform $e_{2}(n)=d^{\prime }(n)-\hat{d}(n),$ i.e., $\;{\hat{\Xi }}_{\text{opt}}=\text{argmin}[E_{2}(\hat{\Xi })],$ where $\hat{d}(n)={\hat{d}}_{1}(n)+{\hat{d}}_{2}(n).$ The extra pulse train estimate ${\hat{d}}_{2}(n)$ is obtained by convoluting an extra pulse unit train estimate ${\hat{u}}_{2}(n)$ with the extra pulse shape estimate ${\hat{r}}_{2}(l),$ i.e., ${\hat{d}}_{2}(n)=\sum_{l}{\hat{u}}_{2}(n)\cdot {\hat{r}}_{2}(n-l),$ where ${\hat{u}}_{2}(n)=\sum_{\mu }{\hat{\xi }}_{\text{opt}}(\mu )\cdot \delta [n-(\mu +0.5)\cdot {\hat{N}}_{0}-\hat{j}(\mu )-\Delta_{\phi }],$ and ${\hat{r}}_{2}(l)$ is obtained via normalized cross-correlation of ${\hat{u}}_{2}(n)$ with the error waveform *e*_1_(*n*), i.e., ${\hat{r}}_{2}(l)=\frac{1}{\sum_{n}{\hat{u}}_{2}(n)}\cdot \sum_{n}{\hat{u}}_{2}(n)\cdot e_{1}(n-l),$ where *l* goes from $-{\hat{N}}_{0}/4+1$ to ${\hat{N}}_{0}/4-1.$

The optimal candidate selection vector ${\hat{\Xi }}_{opt}$ is obtained as follows. $\hat{\Xi }$ is first initialized as a zero vector. Starting with the first pulse, $\hat{\xi }\left(\mu \right)$ is switched to 1 if its current state is 0, and vice versa.The switch is reverted if the error level *E*_2_ does not decreases. After the last pulse is processed, the procedure is restarted. This is repeated until no single new switch yields a decrease of *E*_2_. In a second turn, $\hat{\Xi }$ is initialized as a vector of ones. ${\hat{\Xi }}_{opt}$ is the $\hat{\Xi }$ that minimizes *E*_2_. As a result, $\hat{\xi }\left(\mu \right)$ is 1 at cycle indices *μ* for which extra pulses are detected, and 0 elsewhere.

The proposed approach for estimating the extra pulse train ${\hat{d}}_{2}(n)$ has the advantage over our past peak-picking based approach that no thresholds regarding minimal peak height and minimal peak prominence are necessary. Another advantage of estimating the extra pulse shape via cross-correlation instead of using the shape of the cyclic pulse train is that the height *h* of the extra pulses is estimated implicitly, because ${\hat{r}}_{2}(l)$ is automatically scaled accordingly.

### 3.4 Performance measures and statistical analysis

For each GAW, the detector’s accuracy, and the sum of sensitivity and specificity are determined. The accuracy *Acc* = (*TP* + *TN*)/(*TP* + *TN* + *FP* + *FN*), where *TP* is the number of true positive cycles, i.e., cycles with extra pulses that are detected correctly, *TN* is the number of true negative cycles, i.e., cycles without extra pulses and without detector alarm, *FP* is the number of false positive cycles, i.e., cycles without extra pulses and with false alarm, *FN* is the number of false negative cycles, i.e., cycles with extra pulses that are not detected. The denominator is equal to the number of cycles, i.e., *TP* + *TN* + *FP* + *FN* = *M*. *Acc* can be interpreted as the proportion of cycles that are correctly labelled (with/without extra pulse). For perfect detection, i.e., if no detection errors occur, *Acc* = 1. If the detector behaves randomly, *Acc* converges to an unknown number ≤ max (*ρ*, 1 – *ρ*). Thus, *Acc* is prone to the extra pulse rate. In particular, *Acc* may be very high if extra pulses occur very rarely or very often, even if the detector behaves randomly. In this case, inacceptable sensitivities and specificities may occur that remain unrevealed. This behaviour of the *Acc* limits the interpretation because the parameter *ρ* varies across the GAWs.

The sum of sensitivity and specificity *Se* + *Sp* is obtained as an alternative accuracy measure that is not prone to the parameter *ρ*. The sensitivity *Se* = *TP*/(*TP* + *FN*), and the specificity *Sp* = *TN/*(*TN* + *FP*). For perfect detection, *Se* + *Sp* = 2, while for guessing, *Se* + *Sp* converges to 1.

For analysis of the detector’s robustness, two multiple linear regression models are fit to *Se* + *Sp* with predictors *Irr*, *H*, *ρ*, *h* in the form *Se* + *Sp* = *B*_1_ + *Irr* · *B*_2_ + *H* · *B*_3_ + *ρ* · *B*_4_ + *h* · *B*_5_ [19]. One model is fit for the detector with PSP, and one without. In addition, means and standard deviations of *Acc* and *Se* + *Sp* are obtained, and compared for high and low levels of additive noise as well as high and low irregularity strengths.

For the experiment involving twenty-five bicyclic GAWs, the mean and the standard deviation of only *Se* are reported, because *Sp* is not available due to the inexistence of cycles without extra pulses.

## 4 Results and discussion

Table 2 shows the results of the robustness analysis in terms of linear modelling of the detector performance *Se* + *Sp*. The two detection options, i.e., with and without PSP, are compared. Regarding detection without PSP, negative coefficient estimates reflect that detector performance is adversely affected by increases of the irregularity strength *Irr*, the noise level *H*, and the extra pulse rate *ρ*. This appears to be plausible because irregularity and additive noise limits the detection due to decreases of the signal-to-noise ratio, and the more frequent extra pulses occur, the larger the cross-talk of ${\hat{d}}_{2}(n)$ towards ${\hat{d}}_{1}(n)$ is. In contrast, increases of the extra pulse height *h* affect detector performance advantageously, which is reflected by a positive sign of the coefficient estimate. This appears to be plausible because larger extra pulses are associated with larger signal-to-noise ratios. The same trends are observed when PSP is used, except for the *ρ* parameter (-0.099 versus 0.106). The advantageous effect of *ρ* on the performance of the detector using PSP may be interpreted as a sign that PSP suppresses cross-talk of ${\hat{d}}_{2}(n)$ towards ${\hat{d}}_{1}(n).$

The robustness of the detector using the PSP option is favourable in two parameters, i.e., the irregularity strength *r*, and the extra pulse height *h*. In particular, effects of *Irr* and *h* on the performance when using PSP are half the effects that are observed when no PSP is used. The effect of *h* is non-significant when PSP is used, whereas it is significant without PSP. In other words, small extra pulses are detected equally well as large extra pulses only when PSP is used. However, the detector with PSP is less robust against additive noise than the detector without PSP, which is reflected by an increased coefficient estimate respective *H* (−0.0147 versus −0.00433).

Table 3 summarizes for both detector options the performance measures *Se* + *Sp*, and *Acc*. Means and standard deviations of the four signal classes are shown, i.e., GAWs with small and large energy levels of additive noise (−25 dB cutoff), and GAWs with small and large irregularity strengths (0.1 cutoff). The best performance is observed when PSP is used and *H* ≤ − 25 dB & *Irr* ≤ 0.1 (class I, first row of numbers, right side). A mean *Se* + *Sp* of 1.722 and a mean *Acc* of 0.883 are observed. This result appears to be promising, particularly because this signal class includes GAWs that represent voices with normal to moderately disturbed quality. The other three classes mainly contain GAWs that may be associated with severely disturbed voice quality. When larger energy levels of additive noise are used (class II), the detector without PSP outperforms the one with PSP and achieves a *Se* + *Sp* of 1.445 and an *Acc* of 0.766. At high irregularity strengths and low additive noise levels (class III), the detector with SPS achieves *Se* + *Sp* of 1.287 and an *Acc* of 0.726, which may be acceptable only marginally. For GAWs with high energy levels of noise and large irregularity strengths (last row, class IV), detection appears to be impossible with either of the two detecting options.

Mean sensitivities for detecting extra pulses in subharmonic voices, i.e., with extra pulse rate set to 1, are 29.7% without PSP, and 87.4% with PSP. This observation is plausible because without PSP pulse shapes of the cyclic train may be estimated which are bicyclic, and extra pulses are cancelled out when subtracting the estimate of the cyclic pulse train from the GAW. This adverse effect is successfully tackled when PSP is used, because this strategy ensures that estimated pulse shapes of the cyclic train are single pulsed only.

Assumptions that are needed to be made, limitations of our approach, and differences of the currently presented detector to its previous version are discussed as follows. First, obviously, the used signal model needs to be valid for the signal under test. It is likely that our detector is able to distinguish between phonation with extra pulses and normal voice, but it is not clear how the detector would behave if applied to voice samples with other types of abnormalities, e.g., diplophonic voice, or chaotic phonation. Further testing (and probably training) of the detector will be needed to establish detection that is specific to extra pulses even if other abnormalities occur in the signal. Second, it is assumed that the extra pulses are unjittered and unshimmered, i.e., they occur at fixed times respective the cyclic pulse train’s instantaneous phase, and with fixed heights. These assumptions were relaxed in the past by using a peak-picking based approach [5] to estimate times of extra pulses. However, the current approach has fewer degrees of freedom and appears to be more elegant. Also, we expect that our approach may handle small amounts of extra pulse jitter and shimmer. If large amounts of extra pulse jitter and shimmer occur, it will perhaps become necessary to adapt the detection approach. Third, cross-correlation based segregation of the cyclic pulse train and the extra pulse train relies on the assumption that these trains are uncorrelated. However, we saw in the cyclic pulse train waveform estimate a cross-talk. This cross-talk manifests in the cyclic pulse train as extra pulses, the heights of which depend on the heights of the actual extra pulses and their rate of occurrence. The higher the extra pulses and the higher their rate of occurrence, the larger is the cross-talk. This limitation is tackled successfully in the current approach by introducing PSP to the estimation of the cyclic pulse train, which supresses extra pulses in the cyclic pulse train estimate.

## 5 Conclusion

We propose a synthesizer for GAWs that is capable of adding extra pulses to the cyclic pulse train, and a detector for extra pulses. The detector is tested on 100 synthesized GAWs with random extra pulses, and 25 GAWs with extra pulses in occuring in each quasi-closed phase of the cyclic pulse train known as, bicyclicity, bigeminism, subharmonics, double pulsing, or alternate pulsing. Using signals containing random extra pulses, tests were conducted with different energy levels of additive noise, different strengths of modulation noise, i.e., jitter and shimmer, as well as different extra pulse rates and heights. Two variants of the detector are tested. One detector parameterizes the estimated pulse shapes of the cyclic pulse train using a Chen pulse model, whereas the simpler does not.

Significant steps towards the improvement of our detection approach were made. (i) Our past experience has shown that extra pulses disturb the estimation of the cyclic pulse train, which we successfully tackle with PSP. In particular, a cross-talk had been observed that biased the estimation of the cyclic pulse shape in such a way that it appeared to be double pulsed. We hypothesized that it is possible to suppress cross-talk and thus increase detection performance by using a single-pulse parametric model for the pulses of the cyclic pulse train. Indeed, it is shown experimentally that the detector that uses PSP outperforms the simpler approach if the signals are not corrupted with high energy levels of additive noise. The PSP for cross-talk suppression appears to be particularly relevant for subharmonic voices, because frequent extra pulses result in strong cross-talk without PSP. (ii) Faster candidate selection is proposed for fundamental frequency extraction. (iii) A peak-picking free extra pulse estimator is proposed.

We conclude from our results of robustness analysis that a user of the detector may be given the advice to measure the energy level of the additive noise and irregularity strength before using the proposed detector for extra pulses. Normally, the PSP option should be used, especially if extra pulses occur frequently, as, e.g., in subharmonic voices. If high energy levels of additive noise are observed, the detector should be used without PSP. In cases of high irregularity strengths, the user may be advised not to use the detector with either of the two options.

## Figures and Tables

**Table 1:** Time-invariant synthesis parameters are drawn from the provided distributions. Truncated normal distributions *N*(*μ*, *σ*^2^, *x*, *y*) and uniform distributions *U*(*x*, *y*) are used, where *μ* and *σ* are the means and standard deviations, and *x* and *y* are the lower and upper limits.

Parameter name and symbol		PDF of the distribution
Fundamental frequency	*f_o_*	$𝒩(175,50^{2},50,600)$
Irregularity strength	*Irr*	𝒰(0,0.2)
Open quotient	*OQ*	$𝒩(0.6,0.15^{2},0.1,1)$
Asymmetry	*α*	$𝒩(0.5,0.2^{2},0.1,0.9)$
Opening speed	*S_op_*	$𝒩(0.5,0.2^{2},0.1,0.9)$
Closing speed	*S_cp_*	$𝒩(0.5,0.2^{2},0.1,0.9)$
Extra pulse rate	*ρ*	𝒰(0.1,0.5)
Extra pulse height	*h*	𝒰(0.1,0.5)
Energy level of the additive noise	*H*	𝒰(–50,0)

**Table 2:** Coefficient estimates and p-values of the linear models of the detector performance *Se* + *Sp*. Results are shown for the detector with and without pulse shape parameterization (PSP). The influence of the irregularity strength (*Irr*) on the detection performance decreases by approximately factor 2 (-4.05 versus -2.01) when the PSP option is used. The same is true for the extra pulse height *h* (0.95 versus 0.475). However, the influence of the energy level of the additive noise *H* increases by approximately factor 3 (-0.00433 versus -0.0147) when the PSP option is used. The extra pulse rate *ρ* has no significant effect on the detector performance in either of the options (with or without PSP). n.s.: non-significant.

		Without PSP		With PSP	
			
Predictor	Coefficient	Coefficient estimate	p-Value	Coefficient estimate	p-Value
Intercept	*B*_1_	1.35	< 0.001	0.96	< 0.001
*Irr*	*B*_2_	−4.05	< 0.001	−2.01	< 0.001
*H* (dB)	*B*_3_	−0.00433	0.0235	−0.0147	< 0.001
*ρ*	*B*_4_	−0.099	n.s.	0.106	n.s.
*h*	*B*_5_	0.95	<0.001	0.475	n.s.

**Table 3:** Summary of the means and standard deviations of the performance measures *Se* + *Sp*, i.e., the sum of the sensitivity and specificity, and Acc, i.e., the accuracy. The measures are shown for GAWs with a small level *H* of additive noise, and a small irregularity strength *Irr* (class I signals), GAW with an increased level *H* of additive noise, and a small irregularity strength *Irr* (class II signals), GAWs with a small level *H* of additive noise, and a larger irregularity strength *Irr* (class III signals), and finally GAWs with a large level *H* of additive noise, and a large irregularity strength *Irr* (class IV signals). The best performance achieved the detector using PSP with class I signals (*Se* + *Sp* = 1.722 and *Acc* = 0.883).

			Without PSP		With PSP	
				
			*Se* + *Sp* (mean, std)	*Acc* (mean, std)	*Se* + *Sp* (mean, std)	*Acc* (mean, std)
Signal class	Class I	*H* ≤ - 25 dB & *Irr* ≤ 0.1	1.546, 0.387	0.829, 0.161	1.722, 0.336	0.883,0.153
	Class II	*H* > - 25 dB & *Irr* ≤ 0.1	1.445, 0.346	0.766, 0.168	1.061, 0.237	0.403,0.185
	Class III	*H* ≤ - 25 dB & *Irr* > 0.1	1.136, 0.257	0.716, 0.123	1.287, 0.292	0.726,0.136
	Class IV	*H* > - 25 dB & *Irr* > 0.1	1.067, 0.23	0.652, 0.111	1.067, 0.218	0.432,0.153

## Abbreviations

DFT: discrete Fourier transform
GAW: glottal area waveform
PDF: probability density function
PSP: pulse shape parameterization
RMS: root mean square
SPP: spectral peak picking
